# Cephamycin Resistance in Clinical Isolates and Laboratory-derived Strains of *Escherichia coli,* Nova Scotia, Canada

**DOI:** 10.3201/eid0910.030093

**Published:** 2003-10

**Authors:** Brian Clarke, Margot Hiltz, Heather Musgrave, Kevin R. Forward

**Affiliations:** * Dalhousie University and the Queen Elizabeth II Health Sciences Centre, Halifax, Nova Scotia, Canada

## Abstract

AmpC β-lactamase, altered porins, or both are usually responsible for cefoxitin resistance in *Escherichia coli*. We examined the relative importance of each. We studied 18 strains of clinical isolates with reduced cefoxitin susceptibility and 10 initially-susceptible strains passaged through cefoxitin-gradient plates. Of 18 wild-resistant strains, 9 had identical promoter mutations (including creation of a consensus 17-bp spacer) and related pulsed-field gel electrophoresis patterns; the other 9 strains were unrelated. Nine strains had attenuator mutations; two strains did not express OmpC or OmpF. After serial passage, 8 of 10 strains developed cefoxitin resistance, none developed promoter or attenuator mutations, 6 lost both the OmpC and OmpF porin proteins, and 1 showed decreased production of both. One strain had neither porin alteration or increased AmpC production. Porin mutants may occur more commonly and be less fit and less inclined to spread or cause disease than strains with increased β-lactamase expression.

The development of antibiotic resistance in *Escherichia coli* has important clinical implications. *E. coli* is among the most frequently isolated bacterium in a variety of clinical settings. The development of resistance to older agents such as ampicillin and trimethoprim-sulfamethoxazole, as well as the emerging problem of fluoroquinolone resistance, may substantially limit our antibiotic choices (*1*,*2*).

Although cephamycin-resistant *E. coli* is relatively uncommon, widespread use of β-lactam antiboties may contribute to the development and spread of these strains. In 1999, Sahm et al. reported that 0.16% of *E. coli* were resistant to cephamycins (*3*). At a local level, unpublished data from the Queen Elizabeth II Health Science Centre in Halifax, Nova Scotia indicated that, of the 5,767 strains of *E. coli* processed from urine samples, 0.4% were cephamycin resistant.

All strains of *E. coli* possess a gene that encodes an AmpC β**-**lactamase. Usually, almost no β-lactamase is produced because the gene is preceded by a weak promoter and a strong attenuator (*4*). Surveys of resistance mechanisms in cephamycin-resistant strains have most often identified promoter or attenuator mutations, which results in an up-regulation of AmpC β-lactamase production (*5*–*7*). Occasionally, cephamycin-resistant strains bear mobilized β-lactamases derived from bacteria such as *Citrobacter feundii* (*8*). In addition, mutation or altered expression of outer membrane proteins constituting porins can also contribute to cephamycin resistance. To our knowledge, no investigators have concurrently looked for alterations in porins in addition to promoter-attenuator mutations. Porin alterations might work together to produce a higher level of resistance. In addition, porin alterations may protect *E. coli* and allow subsequent selection for promoter and attenuator mutants.

We examined *E. coli* strains collected at our hospital to determine the basis for resistance. In addition, we created cephamycin-resistant strains of *E. coli* by serial passage on cefoxitin-containing medium to determine which of these two resistance mechanisms was predominant and if our findings were representative of those seen in clinical isolates.

## Materials and Methods

### Bacterial Strains

We collected strains of *E. coli* from midstream urine from inpatients and from patients in the community. Eighteen strains with reduced susceptibility (MIC >8 mg/L) to cefoxitin were included in the analysis, which represented all resistant strains collected during a 6-month period in 2001. For the in vitro development of resistance, we selected 10 clinical isolates from urine that were fully susceptible to β-lactam antibiotics. In both cases, we excluded duplicate strains from the same patient. *E. coli* isolates were identified with conventional biochemical reactions. Organisms were identified by spot indole and β-glucuronidase assays and confirmed by automated Vitek by using GNI+ cards, and antibiotic susceptibilities were performed by using GNS 606 cards (bioMerieux Canada Inc., St. Laurent, Quebec).

### Analysis of Promoter and Attenuator Mutations

*E. coli* chromosomal DNA was isolated with a QIAmp DNA Mini Kit (Qiagen Inc., Valencia, CA), according to the manufacturer’s instructions. Using standard methods, we performed polymerase chain reaction (PCR) with a previously published primer set and protocol which amplifies the region of DNA including the –35 box of the AmpC promoter and the 3′ end of the attenuator, producing a 271-bp amplicon (*5*). Amplification was performed in a PTC-200 Peltier Thermal Cycler (MJ Research, Boston, MA). The amplicons were resolved by 2% agarose gel electrophoresis and visualized after staining with ethidium bromide. The amplicons were purified by using the QIAquick PCR Purification Kit (Qiagen Inc.) and sequenced directly in both directions by using the dideoxy chain termination procedure of Sanger et al. on an ABI Prism automated sequencer at York University Core Molecular Laboratory, Toronto, Ontario, Canada.

### Molecular Fingerprinting

Strains sharing similar promoter or attenuator mutations were fingerprinted by pulsed field gel electrophoresis (PFGE) by using a modification of the method of Gautom (*9*). In brief, a standardized suspension of *E. coli* was prepared from overnight cultures and treated with lysozyme and proteinase K. Plugs were prepared in low-melt agarose. Solidified plugs were deproteinated with sodium lauryl sarcosine and proteinase K, and then washed repeatedly. Two millimeter slices of plug were digested with *Xba*l at 37°C for 3 h in the recommended buffer. Plugs were loaded onto a 1% agarose gel and resolved with a CHEF-Mapper system (Bio-Rad Laboratories, Mississauga, Ontario).

### Outer Membrane Profiles

Bacteria were grown overnight in Luria-Burtani (LB) broth with or without 4 mg/L of cefoxitin. To study Omp expression, 30 mL of LB broth was injected with 300 μL of a bacterial cell suspension from an overnight culture. Cultures were incubated at 37°C in a shaking water bath at 250 rpm to an optical density at 600 nm of 1.0. Cell membranes were disrupted with a sonicator for 2 min with 30-sec cycles intermittent on ice. Cell debris was removed by centrifugation at 10,000 g for 10 min at 4°C. Cytoplasmic membrane proteins were differentially solubilized for 20 min at room temperature with 1.7% sodium-lauryl-sarcosinate in 100 mM Tris, pH 8.0. The suspension was then centrifuged at 100,000 g for 20 min at 4°C, and the pellet containing the outer membrane proteins was resuspended in 100 μL sterile distilled water. Omp preparations were analyzed by urea-sodium dodecyl sulfate-polyacrylamide gel electrophoresis at 30 mA in gels prepared with 11% acrylamide, 0.3% bisacrylamide, 8 M urea, and 0.1% SDS using the discontinuous buffer system of Laemmli (*10*). The gels were stained with Coomassie brilliant blue. The positions of OmpC and OmpF on the Omp profiles were ascertained by comparing the profiles of Omp preparations from the *E. coli* reference strains MH760 (ompR472 OmpC- OmpF+) and MH1461 (envz11 OmpC+ OmpF-) (*11*).

### Development of Cefoxitin-resistant Strains

Cefoxitin-resistant mutants were obtained by serially passaging the wild type strains on 9 cm x 9 cm^2^ gradient plates containing a maximum of 32 mg/L cefoxitin in MH agar, as previously described (*12*). Plates were incubated at room temperature overnight before use to ensure proper diffusion of the antibiotic. Streaked plates were incubated overnight at 37°C and the colony that grew furthest up the cefoxitin gradient was selected and replated on a fresh gradient plate the following day. A total of 12 passages were performed for each strain at a maximum concentration of 32 mg/L cefoxitin and an additional 15 passages at a maximum of 128 mg/L cefoxitin. Isoelectric focusing was performed by using a modification of the method (*13*).

## Results

**Figure 1:**
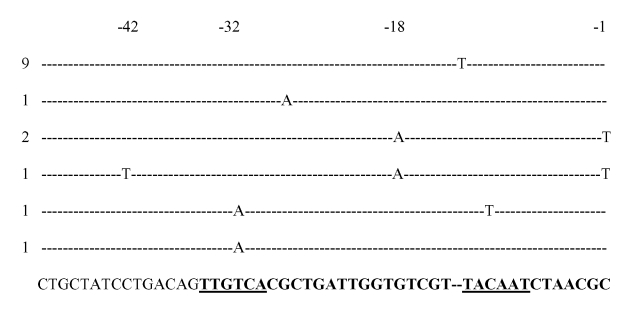
Sequences of *Escherichia coli* AmpC promoters showing mutations detected in cephamycin-resistant strains. The consensus sequence for *E. coli* K12 is shown in the last row. The promoter region is in boldface. The –35 and –10 (Pribnow box) hexamers are underlined. The number of strains with these mutations is indicated on the left.

Each of the 18 strains with reduced susceptibility to cefoxitin was also resistant to ampicillin, cephalothin, and amoxicillin/clavulanate acid. All were imipenem susceptible. Isoelectric focusing demonstrated chromosomal AmpC in all strains; no other β-lactamases were identified. A summary of promoter and attenuator mutations, as well as alterations in outer membrane profiles, is shown in Table 1. Strains QE1–QE9 were identical or closely related by PFGE. Each strain had a 1-bp insertion in the spacer region between the –35 and –10 boxes. This insertion created a consensus 17-bp spacer. In addition to this mutation, these strains had additional mutations at –73, +6, and +81. Strain QE7 also had a deletion in the loop of the attenuator. None of these strains had changes in their outer membrane protein profiles (Figure 1).

**Table 1:** Summary of promoter/attenuator mutations and porin changes in 18 clinical strains of *Escherichia coli*, arranged by pulsed-field type^a^

PFGE type	Strain	MIC (μg/mL)			Patient Location	Promoter mutations	Attenuator mutations	Outer membrane protein profile	
		FOX	CAZ	CRO					
A	QE1	16	<8	<8	HCC	T insertion (–13) to consensus spacer (17 bp)	G to A, right stem; C to T, upstream of stem/loop	No abnormalities	
A1	QE2	8	<8	<8	FD	T insertion (–13) to consensus spacer (17 bp)	C to T, upstream of stem/loop	No abnormalities	
A2	QE3	>32	<8	<8	Inpatient Ward A	T insertion (–13) to consensus spacer (17 bp)	C to T, upstream of stem/loop; C deletion, right stem	No abnormalities	
A2	QE4	16	<8	<8	FD	T insertion (–13) to consensus spacer (17 bp)	C to T, upstream of stem/loop	No abnormalities	
A3	QE5	16	<8	<8	ER	T insertion (–13) to consensus spacer (17 bp)	C to T, upstream of stem/loop; C to T, left stem	No abnormalities	
A4	QE6	>32	<8	<8	HCC	T insertion (–13) to consensus spacer (17 bp)	C to T, upstream of stem/loop	No abnormalities	
A5	QE7	>32	<8	<8	HCC	T insertion (–13) to consensus spacer (17 bp)	C to T, upstream of stem/loop; ATG deletion in loop/right stem (+27 –29)	No abnormalities	
A	QE8	16	<8	<8	FD	T insertion (–13) to consensus spacer (17 bp)	C to T, upstream of stem/loop; G to A, left stem	No abnormalities	
A	QE9	>32	16	<8	Inpatient Ward B	T insertion (–13) to consensus spacer (17 bp)	C to T, upstream of stem/loop; G to A, right stem	No abnormalities	
B	QE10	>32	<8	<8	Inpatient Ward C	C to T (–42); G to A (–18); C to T (–1)	C to A, left stem; C to T, downstream of stem/loop	No abnormalities	
C	QE11	>32	<8	<8	Inpatient Ward C	G to A (–18); C to T (–1)	C to T, downstream of stem/loop	No abnormalities	
D	QE12	>32	<8	<8	FD	G to A (–18); C to T (–1)	C to T, downstream stem/loop	Omp F-	
E	QE13	>32	16	<8	FD	G to A in spacer (–28)	C to T, downstream stem/loop	No abnormalities	
F	QE14	8	<8	<8	ER	T to A (–32), new –35 box; C to T (–11), new –10 box	None	No abnormalities	
G	QE15	>32	16	<8	Inpatient Ward D	T to A (-32), new –35 box	None	Omp C-	
H	QE16	>32	<8	<8	HCC	None	C to T, downstream stem/loop	No abnormalities	
I	QE17	16	<8	<8	FD	None	C to T, downstream stem/loop	No abnormalities	
J	QE18	>32	<8	<8	Inpatient Ward E	None	C deletion, right stem (+31); C to T, downstream stem/loop	No abnormalities	

^a^PFGE, pulsed-field gel electrophoresis; FOX, cefoxitin; CAZ, ceftazidime; CRO, ceftriaxone, HCC, Hants Community Clinic; FD, family doctor’s office; ER, emergency room.

The other nine strains (QE10-QE18) had different PFGE patterns and came from diverse locations (different hospitals and from both outpatients and inpatients). Strain QE10 had a C to T mutation at –42 and a G to A at –18, which created a novel consensus –35. Two strains had the T to A mutation at –32 that is necessary to create the stronger –35 consensus sequence (QE14, 15). Strain QE14 also had the –11 C to T mutation that created the stronger –10 consensus sequence (TACAAT).

Only two strains had no promoter or attenuation loop mutations and no abnormalities of the outer membrane profile (QE16, QE17). Both of these strains had the C to T mutation at position +58 of the attenuator. This mutation would appear not to influence the development of the attenuation loop.

Of the 10 susceptible strains that were serially passaged on gradient plates, 8 developed resistance to cefoxitin (strains LD1–LD5, LD8–LD10). One of these strains had mutations in the AmpC promoter or attenuator regions (Table 2); this strain had two mutations, including a C to T mutation in the left stem of the attenuator, which would result in the transcription of a weak attenuation loop. Both of the mutations were also seen in the initial clinical isolates. This strain also had absent Omp C and Omp F. The remaining cefoxitin-resistant strains either lacked Omp C and Omp F (six strains) or had decreased amounts of OmpC and OmpF (one strain). One strain had no mutations in the promoter or attenuator and normal amounts of Omp C and Omp F.

**Table 2:** Effect of serial passage on cefoxitin gradient on promoter and attenuator regions and outer membrane protein profiles of laboratory wild type *Escherichia coli*^a,b^

Strain	MIC before serial passage (μg/mL)	MIC after serial passage (μg/mL)	Outer membrane protein profile
LD1	<2	>32	None
LD2	<2	>32	Omp C-, Omp F-
LD3	<2	16	Omp C-, Omp F-
LD4	<2	>32	Omp C-, Omp F-
LD5	<2	>32	Omp C-, Omp F-
LD6	<2	8	Not done
LD7	<2	4	Not done
LD8	<2	16	Omp C-, Omp F-
LD9	<2	>32	Omp C- , Omp F-
LD10	<2	>32	Decreased production of OmpC/F

^a^Only those mutations that arose following serial passage are shown. Some parental strains had mutations initially, which were retained in the mutants. ^b^No promoter/attenuator mutations affected β-lactamase production.

## Discussion

**Figure 2:**
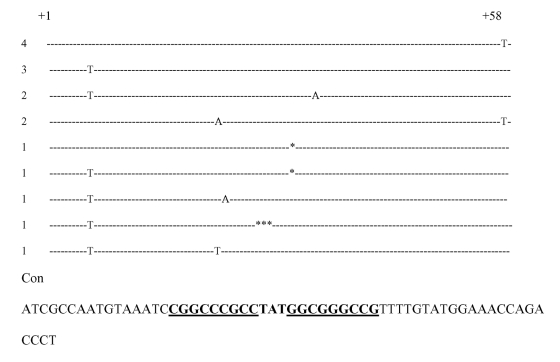
Sequences of *Escherichia coli* AmpC attenuators that were other than the consensus sequence for *E. coli* K12. The hairpin loop is in boldfaced, and the region of the dyad symmetry is underlined. * indicates bp deletion. The number of strains with these mutations is indicated on the left.

The emergence of *E. coli* strains resistant to extended-spectrum cephalosporins and cephamycins should be a cause of concern to clinicians managing infections in both the community and institutional setting. Extended-spectrum cephalosporins and penicillins combined with β- lactamase inhibitors are frequently used for both empirical and definitive treatment of *E. coli* infections. Strains resistant to cephamycins have emerged in recent years. Some of these strains have become resistant by virtue of their hyperproduction of chromosomally encoded AmpC β-lactamases (*5*–*7*). Others have acquired plasmidic β-lactamases; most often those derived from *Citrobacter freundii* (*8*) (Figure 2).

In our survey of *E. coli* strains identified at our center, we found that no strains were resistant as a result of acquired-plasmid–mediated β-lactamases. These strains all produced substantial amounts of AmpC β-lactamase with the isoelectric point characteristic of the *E. coli–*derived AmpC. The predominant reason for the hyperproduction of AmpC appeared to be promoter mutations, attenuator mutations, or both.

Half of the strains appeared to be clonal in origin in that they had the same promoter mutation and the same attenuator mutations. Several of the strains had additional attenuator mutations; however, all of these strains had the same pulsed-field pattern. None of the strains had alterations in their OmpC or OmpF profiles. These strains came from diverse locations within the Queen Elizabeth II Health Sciences Centre complex and from other hospitals. Since we had limited information on the movement of the infected patients within the healthcare system, we are unable to define a common source.

The other nine strains had different pulsed-field patterns. Two strains each had similar promoter mutations, attenuator mutations, or both. Again, these strains came from different locations within our center, the community, and from referring hospitals. Two of these strains had altered outer membrane profiles; one lacked OmpF, the other OmpC. The promoter and attenuator mutations we detected were very remarkably similar to those that had been previously described in France, South Africa, Sweden, and Toronto, Canada. In particular the –42, –31, –18 mutations and spacer inserts have been consistently reported. The –11 promoter mutation that we observed was the second report of this mutation in the *E. coli* AmpC promoter (*14*).

We were able to develop resistance in 8 of 10 strains in vitro. Upon examination, seven of these strains had altered outer membrane protein profiles; six lacked both OmpC and OmpF, and one had decreased production of both of these porin proteins. One strain did not have promoter or attenuator mutations or alterations in the outer membrane protein profile. Sequencing of the promoters and attenuators indicated that only one had mutations that might have affected the amount of β**-**lactamase produced. However, this mutation was present in the wild strain as well.

We found that promoter abnormalities were common in clinical strains but not in lab-generated cefoxitin-resistant isolates. Increased resistance to antimicrobial agents has been shown to be associated with loss of porins in *E. coli* and other gram-negative organisms (*15*–*19*). The major porin proteins of *E. coli*, OmpF and OmpC, are differentially expressed at the transcriptional level by the two-component regulatory proteins, EnvZ and OmpR. The sensory protein, EnvZ, phosphorylates the transcriptional factor OmpR in response to environmental stress. The cellular level of the active form of OmpR, OmpR-phosphate (OmpR-P), is responsible for the differential expression of *ompF* and *ompC* (*20*). Therefore, the tension between the kinase and phosphatase activities of EnvZ controls the level of active OmpR-P in the cell. In the resistant strains selected on gradient plates, we found that most lacked both major outer membrane proteins in comparison to the respective parent strain. The lack of outer membrane protein expression in these isolates could occur by several mechanisms, including loss of kinase activity of EnvZ and mutation of the transcription factor OmpR.

Alternatively, resistance may arise because of decreased pore diameter (*21*,*22*). The in vitro mutant without quantitative changes in Omp C or OmpF or promoter or attenuator mutations may have had this molecular lesion.

We postulate that, while porin deficient mutants are more readily selected by antimicrobial pressure, they are likely less fit. This finding is reflected by the fact that Omp changes are easily created in the laboratory but not found in clinical samples. As a result of their lower fitness, they are more likely to be replaced by wild *E. coli* when antimicrobial pressure has been removed. On the other hand, the widespread clonal dissemination of strains that hyperproduce AmpC by virtue of promoter or attenuator mutations suggests that they are much better able and more likely to contribute to the spread of cephamycin resistance.

The ampC β-lactamase produced by *E. coli* hydrolyzes penicillins, cephalosporins, and cephamycins. In doing so, β-lactamase increases the MICs to third-generation cephalosporins but, as our data suggest, seldom above the breakpoint set by the National Committee for Clinical Laboratory Standards (NCCLS). This situation is analogous to that of many TEM- and SHB-derived β-lactamases. Using third-generation cephalosporins or penicillin/β-lactamase–inhibitor combinations to treat serious infections caused by ampC up-regulated strains may be more imprudent. Just as we would not be inclined to treat an *E. coli* bearing an extended spectrum β-lactamase with a ceftriaxone MIC of 2 mg/L with ceftriaxone, we would not treat an ampC β-lactamase up-regulated *E. coli* strain with a third-generation cephalosporin. To the best of our knowledge, no publication has documented treatment failures in such circumstances, and no NCCLS guidelines exist. Nevertheless, our practice is to report all of these strains as resistant to third-generation cephalosporins and to cautioning against their use.
